# Using Brain-Breaks^®^ as a Technology Tool to Increase Attitude towards Physical Activity among Students in Singapore

**DOI:** 10.3390/brainsci11060784

**Published:** 2021-06-14

**Authors:** Govindasamy Balasekaran, Ahmad Arif Bin Ibrahim, Ng Yew Cheo, Phua Kia Wang, Garry Kuan, Biljana Popeska, Ming-Kai Chin, Magdalena Mo Ching Mok, Christopher R. Edginton, Ian Culpan, J. Larry Durstine

**Affiliations:** 1 Physical Education & Sports Science, National Institute of Education, Nanyang Technological University, Singapore 637616, Singapore; arif20091993@gmail.com; 2 Sports & Physical Education, Singapore University of Social Sciences, Singapore 599494, Singapore; yewcheo@gmail.com; 3 Ministry of Education, Singapore 138675, Singapore; phua_kia_wang@moe.gov.sg; 4 Exercise and Sports Science Programme, School of Health Sciences, Universiti Sains Malaysia, Kubang Kerian 16150, Malaysia; garry@usm.my; 5 Faculty of Educational Sciences, Goce Delcev University, 2000 Stip, North Macedonia; biljana.popeska@ugd.edu.mk; 6 The Foundation for Global Community Health, 1550 W Horizon Ridge Pkwy Ste R #206, Henderson, NV 89012, USA; chinmingkai@yahoo.com; 7 Graduate Institute of Educational Information and Measurement, National Taichung University of Education, 140 Minsheng Road, West District, Taichung City 40306, Taiwan; mmcmok@friends.eduhk.hk; 8 Assessment Research Centre, Department of Psychology, The Education University of Hong Kong, 10 Lo Ping Road, Taipo, N.T., Hong Kong; 9 Department of Health, Recreation and Community Services, University of Northern Iowa, Cedar Falls, IA 50614, USA; christopher.edginton@uni.edu; 10 School of Health Sciences, University of Canterbury, Christchurch 8140, New Zealand; ian.culpan@canterbury.ac.nz; 11 Department of Exercise Science, University of South Carolina, Columbia, SC 29208, USA; ldurstin@mailbox.sc.edu

**Keywords:** video exercises, physical activity, attitudes, online platform, Brain Breaks^®^

## Abstract

The purpose of this study was to investigate the effects of classroom-based Brain Breaks^®^ Physical Activity Solution in Southeast Asia Singaporean primary school students and their attitude towards physical activity (PA) over a ten-week intervention. A total of 113 participants (8–11 years old) were randomly assigned to either an experimental (EG) or a control group (CG), with six classes to each group; the Brain Breaks^®^ group (EG: six classes) and the Control group (CG: six classes). All EG members participated in a Brain Breaks^®^ video intervention (three–five min) during academic classes and the CG continued their lessons as per normal. The student’s attitudes towards PA in both research conditions were evaluated using the self–reported Attitudes toward Physical Activity Scale (APAS), applied before and after intervention. The effects of the intervention on APAS scores were analysed using a mixed model analysis of variance with Time as within-subject and Group as between-subject factors. The analysis revealed evidence in support of the positive effect of classroom video interventions such as Brain Breaks^®^ on student’s attitudes toward benefits, importance, learning, self-efficacy, fun, fitness, and trying to do their personal best in PA. The Brain Breaks^®^ intervention provided a positive significant impact on students in Singapore. This study also revealed that interactive technology tools implemented into the school curriculum benefit students in terms of health and education.

## 1. Introduction

The World Health Organization [1] has defined a person who has a body mass index (BMI) of over 30 kg·m^−2^ as obese, and ≥25 kg·m^−2^ as overweight. Research has concluded that Asians tend to carry a higher percentage of body fat as compared to other racial and ethnic groups of the same BMI [2]. Therefore, the BMI scale for Asians has been lowered (obese: 27.5 kg·m^−2^, overweight: 23 kg·m^−2^). Being overweight and obese during childhood years is linked to chronic diseases risk factors such as diabetes and cardiovascular diseases [3,4]. Furthermore, childhood obesity can persist into adulthood [5,6,7]. Globally, childhood obesity has been on the rise [8].

A possible reason for increased childhood obesity is the availability of current technology. When students use technology, participation in physical activity (PA) is reduced. By the age of 10 years, students have access to at least five different types of screens for viewing at home [9], the use of which is referred to as “screen time”. Significant correlations between the rise of screen time and the lack of PA in students are associated with the rise in obesity [10,11,12,13]. Maher et al. [14] evaluated 2200 Australian students aged 9 to 16 years old and found a high correlation between screen time and the likelihood for a student to be overweight or obese.

The WHO recommends 60 min of daily PA for students 5 to 17 years old [15]. A recent study reported that students are becoming less physically active and more sedentary [16,17]. These trends were established by tracking students PA. Accelerometers were used to measure students’ activity levels during the ages of 6, 9, and 11 years. Students’ PA duration decreased from an average of 66 min a day at age 6 to an average of 53 min a day at age 11 [18]. Jago et al.’s [18] study demonstrated that students spend less time doing PA as they age and are well below WHO’s recommended duration of daily PA. On average, students lost about 63 min of PA weekly at age 11 compared to age 6. Yearly, this decrease equates to 3276 min of lost PA time for the students. The investigation provided additional evidence, suggesting students are becoming less physically active as they grow older. The loss of PA time has likely led to the rise in students’ obesity levels. Jago et al. [18] reported that, at the start of their study, 11% of the students were overweight and 8% were obese. However, by the end of the study, 14% of the students became overweight and 15% were obese. Most students who were overweight or obese at the start of the study remained overweight or obese at the end of the study.

The current generation of students are referred to as “tech-savvy” and show a growing interest in technology. Boone et al. [10] and Lewallen et al. [19] suggest that technology can encourage students to increase their PA levels. Presently, Singaporean students are moving towards digital platforms for learning, gaming, and PA. Singapore educators are actively encouraging the incorporation of technology into lessons or co-curricular activities (CCA) to assist students to cope with the necessary competencies for living in a globalized world. Technology such as HOPSports Brain Break^®^ videos, online streaming, and virtual reality games such as Pokemon GO were developed to increase students’ and adults’ PA time [20]. Althoff et al. [21], focusing on the influence of Pokemon Go on PA levels, found a significant increase in PA by sedentary users when starting to play using this particular form of technology. Results were calculated by tracking the number of steps the user took before, during, and after playing a game. However, no significant difference was found in users who were already physically active.

Exercise videos such as HOPSports and JumpJam are becoming more popular with students. The current study specifically selected HOPSports Brain Breaks^®^ videos, as these videos utilize a dynamic online platform that is closely aligned to the Whole School, Whole Community, and Whole Child (WSCC) Guidelines [22,23], and the United Nations Sustainable Development Goals (UNSDG) [24,25]. Singapore, a small country with limited land and lack of natural resources, recognizes the challenges of sustainable development. Prime Minister Lee Hsien Loong stated that Singapore is committed to the 2030 Agenda for Sustainable Development [26]. Further, the UNSDG statement encompasses social–emotional learning, nutritional education, PA and education, career education, and environmental education all into one online platform. These values are also in-line with Singapore’s Ministry of Education’s (MOE) Desired Outcomes of Education and 21st Century Competencies [27]. All classrooms in local schools are equipped with internet connections, a desktop computer, a sound system, and a projector or interactive whiteboard to ensure that students and teachers are able to keep abreast with advancements in technology.

Brain Breaks^®^ videos are video exercises which average three–five min duration. Previous studies concluded that the use of Brain Breaks^®^ videos help develop positive changes in students’ attitudes towards PA [28,29,30,31,32]. Students who completed the intervention did simple aerobic/movement exercises following the video instructions. Also included in these videos was content pertaining to health and nutrition, social learning, character building, and arts and culture [24,33]. The results of these studies indicated a positive change in the intervention group’s attitudes and interest towards PA. Krause & Benavidez [34] found that technology presented a more effective way to promote PA as compared to the traditional games and sports. As Singapore is a highly developed country, technology leverage is a more effective way to promote school students’ PA. Brain Breaks^®,^ as an intervention tool, has already been shown to improve students’ knowledge, self-awareness, and positive attitude leading towards motivation for increased PA [35].

Thus, the aim of this study was to investigate the use of Brain Breaks^®^ videos and the videos’ effects on Singaporean students’ attitudes towards PA and possible increasing PA participation. This study is the first to examine the use of Brain Breaks^®^ videos in the context of Singapore’s students and their school system. We hypothesize that Brain Breaks^®^ videos will positively impact students’ attitudes, which may increase PA participation.

## 2. Materials and Methods

### 2.1. Research Design

This study was a two-group (experimental/control) quasi-experimental design. The experimental group (EG) participated in the Brain Breaks^®^ intervention program of performing the Brain Breaks^®^ video for 10 weeks, averaging three–five min daily during their class time, five days per week. The Brain Breaks^®^ video was projected on a screen using a projector in the classroom. The videos featured physical movement activities, accompanied with songs and dance, and movements that can be done safely by maintaining adequate social distance between students (the full content of the program can be retrieved at Kuan et al. [24]. Students were invited to follow the movements shown on the screen. To maintain students’ enjoyment and motivation, a variety of videos were played for each of the five days. Online access to the official project website is found at https://brain-breaks.com, (accessed on 1 March 2019) [17,24]. The control group (CG) continued their academic lessons as per normal for 10 weeks consecutively without video intervention. Participants’ attitudes toward PA in both groups were measured before and after the intervention using the self-reported Attitudes toward Physical Activity Scale (APAS) questionnaire. Data collection took place before the 10-week intervention in the first week of the school term, and again at the end of the intervention. Participants were obtained from 12 student classes from a local Singapore school system. These 12 intact classes were randomly assigned into either the EG (six classes) or the CG (six classes) groups using a computer-generated randomization (www.randomization.com, accessed on 1 March 2019).

### 2.2. Ethical Approval

This study obtained approval from the Institutional Review Board from Nanyang Technological University (NTU-IRB Reference Number-2019-01-025), and was a school collaborative research initiative. Parents and students voluntarily signed informed consent forms agreeing to participate in this study.

### 2.3. Participants

Participants comprised 113 (47 boys, 66 girls) clinically healthy students ranging from 8 to 11 years old (Table 1). According to the intact class to which the student belonged, the classes were separated into either the experimental Brain Breaks^®^ group (EG: six classes of 48 total students) or the Control group (CG: six classes of 65 total students). All students in the recruited classes were invited to participate. Students with prior injuries or conditions such as heart problems were excluded. Students who were excused from physical activity as advised by their doctors or had not acquired parental consent were also excluded. The required sample size was estimated using G-Power Version 3.1. Based on the repeated measures ANOVA with two research conditions (experimental and control group) ×2 time points (baseline, and post), statistical power set at 80% with a 95% confidence interval, and an effect size of 0.25 [17], a sample size of 98 was calculated. With a 15% dropout, a total of 113 was judged to be sufficient to detect the hypothesized between-condition differences.

### 2.4. Measures

#### Students’ Attitudes toward Physical Activity Scale (APAS)

The APAS is a self-reported questionnaire used to measure beliefs, attitudes, and self-efficacy towards PA from students. The questionnaire is composed of seven sections using Likert-type scales. An additional section gathers demographic information regarding gender, age, school grade level, body height, and weight. The remaining seven sections referred to: (F1) ‘promoting holistic health’, 10 items constructed to measure students’ attitudes toward the effectiveness of physical activities to promote holistic health. An example item is, “Being physically active helps to give me good health”; (F2) ‘importance of exercise habit’: five items designed to measure attitudes toward the importance of doing exercise as a lifestyle. An example item is, “It is important to be physically active for my health”; (F3) ‘self-efficacy in learning with video exercises’: 11 items to measure self-efficacy in learning curriculum content by using video exercises. Example items are, “I learn about art through exercise videos,” and “I know how to do physical activity if there is an exercise video to follow”; (F4) ‘self-efficacy in selecting video exercises for themselves’: four items to measure a student’s level of independence when performing their self-selected exercise video. An example item is, “I know how to choose physical activity in the exercise video that suits me.” (F5) ‘exercise motivation and enjoyment’: 14 item scale designed to measure motivation and enjoyment when doing physical exercise. An example item is, “I think physical activity is fun”; (F6) ‘self-confidence on physical fitness’: eight items constructed to measure self-perception of physical fitness. An example item is, “I am confident with my balance”; (F7) ‘trying to do my personal best’: five items constructed to measure personal best goal orientation to engage in PA. An example item is, “My target is to go beyond what I have achieved in physical activity”.

Questionnaire response options for each item were a four point Likert-type response category including “strongly disagree”, “disagree”, “agree”, and “strongly agree”. The seven scales in the original version of this questionnaire were validated for their reliabilities, uni-dimensionality, effectiveness of the response categories, and absence of gender differential item functioning (DIF) by Mok et al. [30] using the Rasch analysis. A subsequent study by Dinc et al. [31] updated the questionnaire and further enhanced internal consistency. The current study reported here made use of the updated version of APAS. The Cronbach’s Alpha reliability coefficients for Singapore students ranged from 0.81 to 0.92 (Table 2).

### 2.5. Data Analysis

Data analysed were completed using the IBM SPSS version 26.0 software (IBM Corp., Armonk, NY, USA). Distribution of the data including mean (M) + standard deviation (SD) of the variables was assessed for normality (Skewness and Kurtosis values were close to 0 and z-values ranged between –1.96 and 1.96). No non-normal distributions were identified. Effects of applied Brain Breaks^®^ intervention on APAS scores were analysed using a two-way 2 × 2 mixed analysis of variance (ANOVA) with Time (pre-test/ post-test) as the within-subject factor (repeated measures) and Group (EG/CG) as the between-subject factor. The partial eta-squared (η^2^) effect sizes for the tests were calculated to indicate the magnitude of the effects. The level of statistical significance was set as *p* < 0.05.

## 3. Results

A one-way ANOVA Brown–Forsythe test was used to compare pre-test between groups. As presented in Table 3, where CG indicated higher scores; pre-F4 (EG: 3.02 ± 0.62 vs. CG: 3.10 ± 0.60, *p* < 0.001), pre-F6 (EG: 2.97 ± 0.73 vs. CG: 3.05 ± 0.73, *p* < 0.001), pre-F7 (EG: 3.20 ± 0.61 vs. CG: 3.29 ± 0.65, *p* < 0.001). Table 3 presents the mean scores of the APAS obtained by EG and CG groups before and after intervention, the results of the 2 × 2 mixed ANOVA with one within-subject factor (Time: before or after intervention) and one between-subject factor (Group: experimental or control), as well as the effect sizes (Time η^2^, Group η^2^ and Time*Group η^2^). A significant increase in the mean scores of all APAS scales, for EG and CG, between pre-and post-10-weeks intervention was found (Table 3). The main Time effect was significant (*p* < 0.05) for all APAS scales. Effect sizes of the Time factor ranged from 0.04 to 0.19.

The results found in Table 3 revealed significant main effects on Group for importance (*p* = 0.012), learning (*p* < 0.001), self-efficacy (*p* < 0.001), fun (*p* = 0.034), and fitness (*p* = 0.022) scales. Effect sizes for the Group main effect were relatively small (η^2^ < 0.12) for all scales, except for Learning (η^2^ = 0.23).

**Figure 1:**
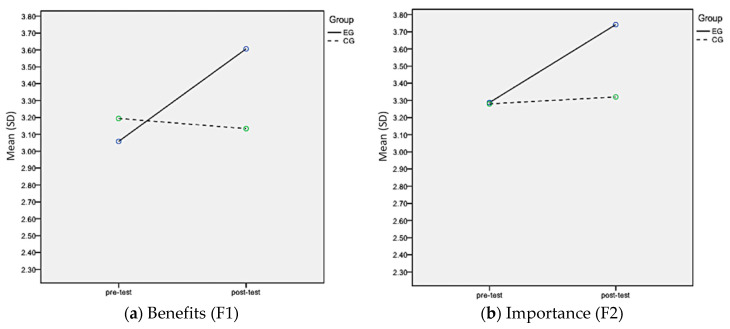

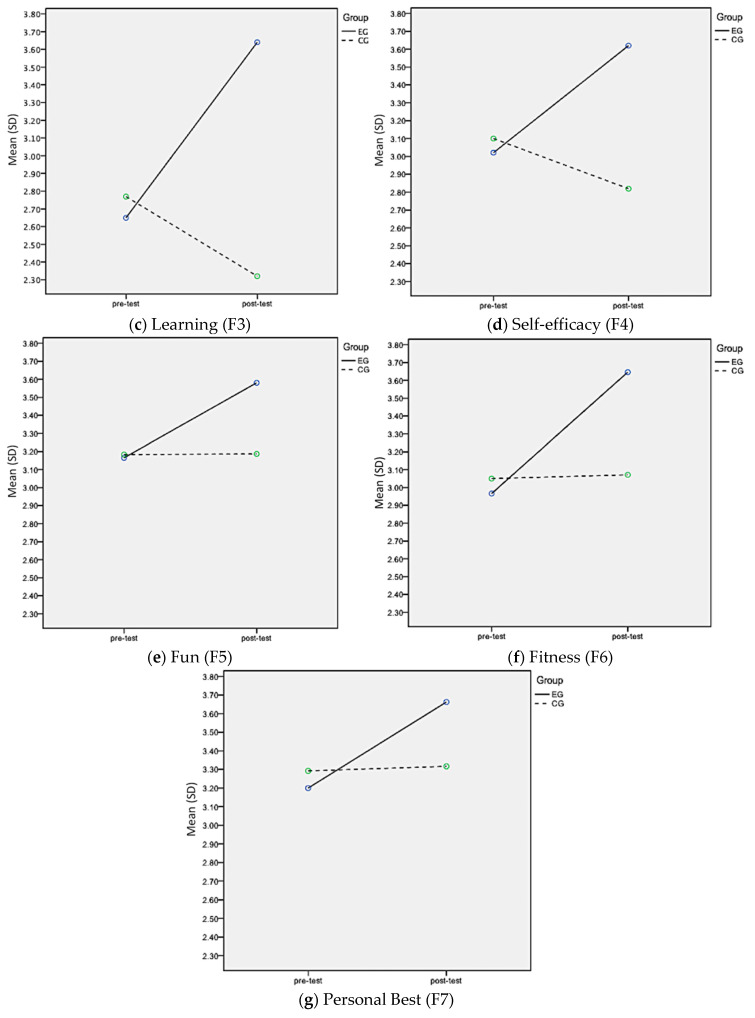
Scale Mean Values of the Experimental and the Control Groups at Pre-test and Post-test.

**Figure 2:**
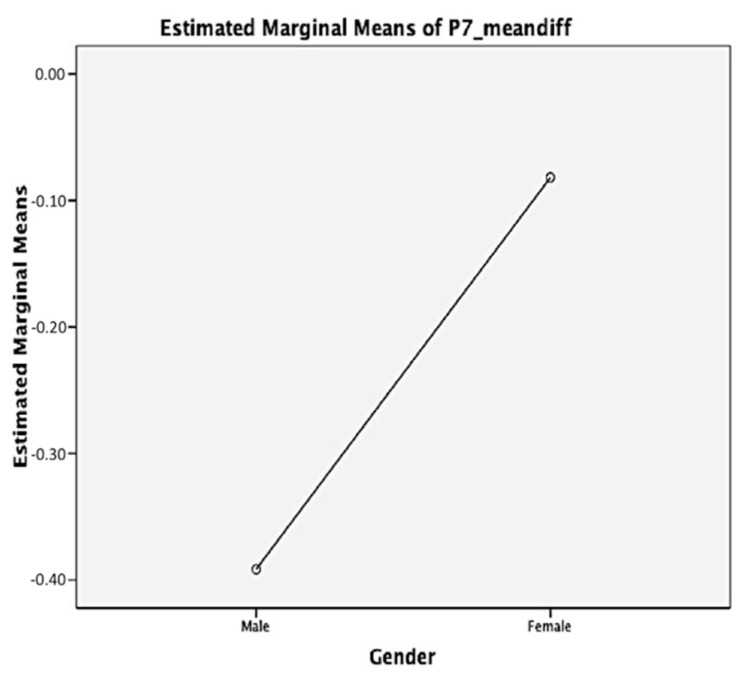
Post-Self–efficacy Scores in Learning Curriculum Subjects through Video Exercises of the Experimental Group.

Time*Group interaction effects were significant for all APAS scales (*p* < 0.05) (Table 3). Effect sizes of the Time*Group interaction effect ranged from 0.08 (Personal Best scale) to 0.37 (Learning scale). EG showed an increase for all pre-test to post-test APAS scale scores when compared to CG (Figure 1). The one-way ANOVA Brown–Forsythe test was also used to compare between genders for EG. A significant difference was found for post-F3, where boys, when compared to girls, had higher self-efficacy scores in learning curriculum subjects through video exercises (EG boys: 3.76 ± 0.28 vs. EG girls: 3.54 ± 0.39, *p* = 0.04) (Figure 2).

## 4. Discussion

The purpose of this study was to determine whether using Brain Breaks^®^ videos would positively change Singaporean students’ attitudes towards PA and increase their PA participation. Empirical evidence in support of the positive effect of Brain Breaks^®^ videos of three–five min a day, five times a week for 10 weeks, to enhance students’ attitude towards PA was found. This study is the first conducted in Southeast Asia among Singaporean students. The findings from this study are similar to the findings from previous studies investigating the impact of Brain Breaks^®^ videos on students aged 9 to 11 years old in China [25], Malaysia [16], Turkey [31], Lithuania [33], Poland [28] and Macedonia [32], students 12 years old [36], and students in higher education [31]. These previous studies found that students utilizing Brain Breaks^®^ videos had positive changes in their attitudes towards PA. This difference could be due to the fun element, with movement and music, found in the exercise videos that the students performed during their short breaks. In addition, no significant differences were found between the scores of the experimental and control groups before the intervention. These earlier studies report that short amounts of exercise completed in the classroom are linked to increases in students’ on-task behavior and PA [28,33], improved self-awareness, self-efficiency in using video exercises [32], fun, and effort to do their personal best [25] while participating in Brain Break^®^ activities. Other studies have shown that students who are more physically active perform better in their academic studies [37,38]. One potential explanation for better academic achievement is that PA increases the blood flow to the brain and may be the link to better academic performance. With more blood flowing to the brain, oxygen delivery is increased, which potentially has a positive impact on brain function [38].

As the results from the current study show a positive increase in Singapore students’ attitudes towards PA, Singapore school systems should consider the integration of daily Brain Breaks^®^ into the school curriculum. This internet format is stable and requires state-of-the-art infrastructure (e.g., projectors, sound system, etc.) and technology (e.g., internet, fast wireless network, etc.). Utilizing such a system would assist students during long periods of continuous lessons where teachers and students can experience intellectual or mental fatigue that can reduce their ability to focus and concentrate. Short PA breaks between lessons would help teachers and students to refocus for the next lesson. Alternatively, Brain Breaks^®^ videos can be integrated prior to the end of the students’ recess or snack time.

In 2014, Singapore launched a project named the Smart Nation Initiative [39] to address topics such as health/well-being. Experiences gained from this collaboration enabled Singapore to grow and move the city’s economy towards greater technology for services-based applications. These cooperative relationships allowed Singapore to make significant gains towards their smart and sustainable city goals by creating a system that provided the backbone to achieve other sustainable goals. These goals are, in part, attained by using either free or affordable high-speed internet. Globally, cities need to follow the example of Singapore in using advanced systems to achieve educational sustainability goals. Presently, almost every Singaporean household has access to the internet, and almost every student owns at least one technological device (e.g., smart phone, desktop, tablets, etc.). Hence, students are able to access the Brain Breaks^®^ videos at home and carry out PA independently.

With the recent COVID-19 pandemic, the Ministry of Education (MOE) developed home-based learning (HBL) for all schools. Students can login into the school’s system and attend online lessons. As students become accustomed and proficient with HBL, they can access Brain Breaks^®^ videos. Hence, future studies can investigate whether greater access to exercise videos leads to increased students’ motivation for learning while simultaneously keeping them physically active. Online PA programs do increase PA accessibility, are available, and should be used during and after the COVID-19 pandemic [40,41,42,43,44,45]. In this regard, development of online programs is needed, and these programs must be consistent with the United Nations Sustainable Development Goal 3 (Health and Well-Being) and Goal 11 (Sustainable Cities and Communities) [42,45,46,47].

Effective implementation of classroom-based PA intervention is highly dependent on the cooperation and interaction of children and teachers throughout the intervention [17]. The education system in Singapore is based on a systematic design and structured timetable. Furthermore, the teachers’ creativity, behavior, cooperation, and personal motivation are essential in implementing the program. The teachers’ knowledge about the benefits and advantages of the program do play an important role in the students’ motivation. In addition, the school principal’s support for the program is also an important factor in monitoring and ensuring that the students carry out the Brain Breaks^®^ PA program in a safe environment.

The self-reported nature of APAS is one study limitation. For future studies, APAS could be supplemented by using accelerometers or pedometers to track the students’ PA level. The added information from these measures would provide quantifiable data pertaining to students’ PA, and provide insights into the physical responses of students other than their perceived attitudes toward PA. Another possibility is that the positive increase in student attitude to PA found in this study was from their Physical Education portion of the schools’ curriculum. Therefore, future studies could evaluate the interaction of Brain Breaks^®^ and current physical education curriculums.

Besides measuring attitudes toward PA that affect behavior and academic achievement, other variables such as changes in fundamental movement and motor abilities, students’ effects on-time reaction, and sense of rhythm are also important elements that can be incorporated as part of future investigations to improve Brain Breaks^®^ videos. This study is the first to assess the effects of Brain Breaks^®^ on Singapore’s primary school children. This acknowledgement, along with the study’s experimental design and implementation in a real-world classroom setting, are distinct study strengths. These findings provide compelling evidence for the potential use of classroom-based PA and increased awareness of technological solutions that can increase Singapore’s children PA engagement.

## 5. Conclusions

The results of this Southeast Asian study provide further international evidence on the positive benefits of Brain Breaks^®^ Physical Activity Solutions on students’ learning in support of evidence found by other international studies completed in China, Malaysia, Poland, North Macedonia, South Africa, and Turkey. This study adds to the understanding that investing as little as three–five min a day is enough time to improve students’ perception of PA and subsequently lead students to engage in more PA during their free time. What remains unclear is whether these behaviors are carried into adulthood. From this study comes the recommendation to utilize and implement exercise videos as an interactive technology tool in the Singapore school system curriculum. The addition of exercise videos will benefit students in terms of enhancing their health and facilitating the education process.

## Figures and Tables

**Table 1:** General Characteristics of the Participants (*n* = 113, boys = 47, girls = 66).

Variables	Total (*n* = 113) Mean ± SD	EG (*n* = 48) Mean ± SD	CG (*n* = 65) Mean ± SD
Gender			
Male (*n*, %)	47 (41.6%)	22 (45.8%)	25 (38.5%)
Female (*n*, %)	66 (58.4%)	26 (54.2%)	40 (61.5%)
Age (years)	9.68 ± 0.95	9.71 ± 0.99	9.66 ± 0.94
Height (m)	1.38 ± 8.27	1.37 ± 0.09	1.39 ± 0.09
Weight (kg)	35.21 ± 10.21	34.91 ± 10.97	35.43 ± 10.55
Body Mass Index (kg·m^−2^)	18.19 ± 2.86	18.24 ± 4.18	18.16 ± 3.67

Note. EG = Experimental Group, CG = Control Group; Age (years); Height (meters, m); Weight (kilograms, kg); Body Mass Index (kilograms per meter square, kg·m^−2^). No significant difference between EG and CG was found for all variables above (*p* > 0.05).

**Table 2:** Cronbach’s Alpha Reliability Analysis of APAS for Singapore Students (*n* = 113).

Scale	Number of Items	Cronbach’s Alpha
Promoting Holistic Health (F1)	10	0.84
Importance of Exercise Habit (F2)	5	0.81
Self-efficacy in Learning with Video Exercises (F3)	11	0.93
Self-efficacy in Selecting Video Exercises (F4)	4	0.90
Exercise Motivation and Enjoyment (F5)	14	0.87
Self-confidence on Physical Fitness (F6)	8	0.92
Trying to do Personal Best (F7)	5	0.82

**Table 3:** Descriptive Statistics and ANOVA at pre-test/post-test for students in the Experimental Group (EG; *n* = 48) and Control Group (CG; *n* = 65).

Variables on Physical Activity	Group	Pretest	Posttest	Time			Group			Time*Group		
		Mean (SD)	Mean (SD)	*F*	*p*	η^2^	*F*	*p*	η^2^	*F*	*p*	η^2^
Benefits (F1)	CG	3.19 (0.55)	3.13 (0.64)	18.87	<0.001	0.15	3.86	0.052	0.03	29.29	<0.001	0.21
	EG	3.06 (0.51)	3.61 (0.37)									
Importance (F2)	CG	3.28 (0.61)	3.32 (0.60)	17.89	<0.001	0.14	6.49	0.012	0.06	12.57	0.001	0.10
	EG	3.29 (0.52)	3.74 (0.31)									
Learning (F3)	CG	2.77 (0.78)	2.32 (0.88)	9.35	0.003	0.08	32.55	<0.001	0.23	66.08	<0.001	0.37
	EG	2.65 (0.67)	3.64 (0.36)									
Self-efficacy (F4)	CG	3.10 (0.60)	2.82 (0.74)	4.38	0.039	0.04	14.89	<0.001	0.12	33.45	<0.001	0.23
	EG	3.02 (0.62)	3.62 (0.51)									
Fun (F5)	CG	3.18 (0.58)	3.19 (0.66)	13.70	<0.001	0.11	4.59	0.034	0.04	13.27	<0.001	0.11
	EG	3.17 (0.48)	3.58 (0.38)									
Fitness (F6)	CG	3.05 (0.73)	3.07 (0.69)	25.92	<0.001	0.19	5.42	0.022	0.05	22.89	<0.001	0.17
	EG	2.97 (0.73)	3.65 (0.39)									
Personal Best (F7)	CG	3.29 (0.65)	3.32 (0.63)	11.59	<0.001	0.10	2.08	0.153	0.02	9.36	0.003	0.08
	EG	3.20 (0.61)	3.66 (0.43)									

Note. EG = Experimental Group, CG = Control Group. * Time and group interactions.

## Data Availability

Data is available upon request from the corresponding author.
